# Dried Blood Spot Postmortem Metabolic Autopsy With Genotype Validation for Sudden Unexpected Deaths in Infancy and Childhood in Hong Kong

**DOI:** 10.7759/cureus.62347

**Published:** 2024-06-13

**Authors:** Ling Yin Hung, Chloe M Mak, Ka Chung Foo, Chun Hei Toby Chan, Hok-Fung Tong, Tsz Ki Wong, Hoi Shan Leung, Ka Chai Cheung, Han Chih Hencher Lee, Chor Kwan Ching

**Affiliations:** 1 Department of Pathology, Princess Margaret Hospital, Hong Kong, CHN; 2 Department of Pathology, Hong Kong Children’s Hospital, Hong Kong, CHN; 3 Forensic Pathology Service, Department of Health, Hong Kong, CHN

**Keywords:** sudden infant death syndrome (sids), multiple acyl-coa dehydrogenase deficiency, glutaric aciduria type ii, molecular autopsy, metabolic autopsy, inborn errors of metabolism, sudden unexpected infant death, sudden unexpected death

## Abstract

Background

Inborn errors of metabolism (IEM) are collectively rare but potentially preventable causes of sudden unexpected death (SUD) in infancy or childhood, and metabolic autopsy serves as the final tool for establishing the diagnosis. We conducted a retrospective review of the metabolic and molecular autopsy on SUD and characterized the biochemical and genetic findings.

Methodology

A retrospective review of postmortem metabolic investigations (dried blood spot acylcarnitines and amino acid analysis, urine metabolic profiling where available, and next-generation sequencing on a panel of 75 IEM genes) performed for infants and children who presented with SUD between October 2016 and December 2021 with inconclusive autopsy findings or autopsy features suspicious of underlying IEM in our locality was conducted. Clinical and autopsy findings were reviewed for each case.

Results

A total of 43 infants and children aged between zero days to 10 years at the time of death were referred to the authors’ laboratories throughout the study period. One positive case of multiple acyl-CoA dehydrogenase deficiency was diagnosed. Postmortem reference intervals for dried blood spot amino acids and acylcarnitines profile were established based on the results from the remaining patients.

Conclusions

Our study confirmed the importance of metabolic autopsy and the advantages of incorporating biochemical and genetic testing in this setting.

## Introduction

Many inborn errors of metabolism (IEM) are known to present with sudden unexpected death (SUD) in infancy and childhood. Well-known examples include fatty acid oxidation defects, urea cycle disorders, organic acidurias, and disorders of galactose and fructose metabolism [1]. It was estimated from previous studies that metabolic disorders account for 3-5% of SUD cases [2,3]. Often these patients remain asymptomatic and well before the acute fatal decompensation of their metabolic disorder. As many of these conditions are treatable if diagnosed early, expanded newborn screening for IEM has been advocated worldwide as a cost-effective screening approach and has been implemented in our locality stepwise since 2015. In 2020, a government-funded newborn screening program for a panel of 26 IEMs, including disorders of amino acid, disorders of organic acids, disorders of fatty acid oxidation, and other IEMs, was implemented in all public hospitals with maternity services [4]. The service, however, does not cover babies born in the private sector. Nevertheless, metabolic investigations have long been recommended as part of routine postmortem examination for SUDs in infancy and childhood by various guidelines and protocols [1,5]. Recommended tests include acylcarnitine and amino acid analysis by tandem mass spectrometry on dried blood spot and bile specimens [1,6-10], organic acid analysis in urine or vitreous specimens [11], as well as enzymatic and other functional studies on tissue specimens such as skin, muscle, and liver biopsy [3,10,12]. Recently, the use of massively parallel sequencing in postmortem DNA samples has also been reported as an alternative diagnostic approach [13,14].

The previous local report on the causes of unexpected deaths in children under two years of age was published in 2006 [15]. IEM was identified as the cause of death in five cases (2.7%) out of 183 cases of sudden deaths recorded throughout the four-year study period between 1997 and 2002. In 2010, our group also encountered a previously well 14-year-old Chinese boy who presented with SUD in his adolescence [16]. Postmortem investigations by biochemical and molecular testing confirmed the diagnosis of multiple acyl-CoA dehydrogenase deficiency, or glutaric aciduria type II. Cascade family screening enabled accurate risk assessment in other family members and avoided unnecessary treatments in unaffected members. The importance of postmortem metabolic investigations was again exemplified by this case. Thus, in 2016, our group began providing metabolic investigation to infants and children presenting with SUD. Postmortem samples for metabolic investigations were collected at autopsy in selected cases with inconclusive autopsy findings or patients with autopsy findings suggestive of a metabolic disorder, such as fatty infiltration of the liver. Tests provided included dried blood spots for acylcarnitines and amino acid analysis, urine organic acids, metabolic profiling, and next-generation sequencing with an IEM gene panel. This study aims to retrospectively review the results of all postmortem metabolic investigations performed at our laboratories to improve the understanding of the local epidemiology and establish reference intervals for the interpretation of acylcarnitines and amino acids on postmortem dried blood spots.

## Materials and methods

Subjects and materials

All cases of infants and children under the age of 18 who presented with SUD in Hong Kong between October 2016 and December 2021 referred to the authors’ laboratories for metabolic investigations were retrospectively reviewed. Clinical notes and perimortem laboratory investigations on initial presentation where available were retrieved from hospital electronic patient records. Autopsy records and results of postmortem laboratory investigations, including metabolic analyses, were retrieved from laboratory databases.

This study was approved by the Hospital Authority Kowloon West Cluster Research Ethics Committee (KW/EX-21-060(158-03)), Hong Kong Children’s Hospital Research Ethics Committee (HKCH-REC-2021-052), and Ethics Committee of the Department of Health (L/M 110/2021) (complying with local research ethical review regulation based on seven hospital clusters and the Department of Health).

Methods

Postmortem dried blood spot samples collected on Whatman 903 filter paper were subjected to amino acid and acylcarnitines analyses using the Neobase non-derivatized MSMS Kit (Perkin Elmer, Waltham, MA, US) on the Waters Acquity TQD LC-MS/MS system (Waters, Milford, MA, US). A dried blood disk (3.2 mm in diameter) was punched out for extraction and the eluate was injected into liquid chromatography-mass spectrometry (LC-MS)/mass spectrometry (MS) directly without derivatization and chromatography. The target amino acids and acylcarnitines were detected and quantified using the multiple reaction monitoring mode. Urine multi-target metabolic profiling was performed with an in-house dilute-and-shoot LC-MS/MS protocol using gradient high-pressure liquid chromatography (Agilent Zorbax Eclipse AAA 4.6 mm × 15 cm, 5 µm reversed-phase column) and electrospray ionization on the Waters Xevo TQ-MS System (Waters, Milford, MA, US). The method allows simultaneous analyses of selected organic acids, acylglycines, acylcarnitines, amino acids, purines, pyrimidines, and other relevant markers in IEM by multiple reaction monitoring analysis. Next-generation sequencing was performed on the genomic DNA extracted from the dried blood spot specimen using a custom amplicon-based IEM panel on the iSeq Sequencing System (Illumina, Inc, San Diego, CA, US), details of which had been previously published [17]. Data analysis was limited to 75 genes (Table 1) which are causative of conditions that may present with SUD. Pathogenicity of variants was classified according to the standards and guidelines for the interpretation of sequence variants published by the American College of Medical Genetics and Genomics in 2015 [18].

**Table 1:** List of genes analyzed for the sudden unexpected infant and child death cases.

Genes		MIM	Condition	MIM
1.	ACAD8	604773	Isobutyryl-CoA dehydrogenase deficiency	611283
2.	ACAD9	611103	Mitochondrial complex I deficiency, nuclear type 20	611126
3.	ACADM	607008	Medium-chain acyl-CoA dehydrogenase deficiency	201450
4.	ACADS	606885	Short-chain acyl-CoA dehydrogenase deficiency	201470
5.	ACADSB	600301	2-methylbutyrylglycinuria	610006
6.	ACADVL	609575	Very-long-chain acyl-CoA dehydrogenase deficiency	201475
7.	ACAT1	607809	Alpha-methylacetoacetic aciduria (alternative titles: beta-ketothiolase deficiency/2-methyl-3-hydroxybutyric academia/mitochondrial acetoacetyl-CoA thiolase deficiency)	203750
8.	ACAT2	100678	Acetyl-CoA acetyltransferase-2 deficiency	614055
9.	ACSF3	614245	Combined malonic and methylmalonic aciduria	614265
10.	AHCY	180960	Hypermethioninemia with deficiency of S-adenosylhomocysteine hydrolase	613752
11.	ALDH4A1	606811	Hyperprolinemia, type II	239510
12.	ALDH6A1	603178	Methylmalonate semialdehyde dehydrogenase deficiency	614105
13.	AMT	238310	Glycine encephalopathy (non-ketotic hyperglycinemia)	605899
14.	ARG1	608313	Argininemia	207800
15.	ASL	608310	Argininosuccinic acidemia	207900
16.	ASS1	603470	Citrullinemia type I	215700
17.	AUH	600529	3-methylglutaconic aciduria, type I	250950
18.	BCKDHA	608348	Maple syrup urine disease, type Ia	248600
19.	BCKDHB	248611	Maple syrup urine disease, type Ib	248600
20.	BTD	609019	Biotinidase deficiency	253260
21.	CBS	613381	Homocystinuria	236200
22.	CPS1	237300	Carbamoylphosphate synthetase I deficiency	608307
23.	CPT1A	600528	Carnitine palmitoyltransferase type I deficiency	255120
24.	CPT2	600650; 608836	Carnitine palmitoyltransferase II deficiency	600649
25.	DBT	248610	Maple syrup urine disease, type II	248600
26.	DECR1	222745	2,4-dienoyl-CoA reductase deficiency	616034
27.	DLD	238331	Maple syrup urine disease, type III (dihydrolipoamide dehydrogenase deficiency)	246900
28.	ETFA	608053	Glutaric acidaemia type II	231680
29.	ETFB	130410	Glutaric acidaemia type II	231680
30.	ETFDH	231675	Glutaric acidaemia type II	231680
31.	ETHE1	608451	Ethylmalonic encephalopathy	602473
32.	FAH	613871	Tyrosinaemia type I	276700
33.	GCDH	608801	Glutaric acidemia type I	231670
34.	GCH1	600225	Dystonia, DOPA-responsive, with or without hyperphenylalaninemia	128230
	GCH1	600225	Hyperphenylalaninemia, BH4-deficient, B	233910
35.	GCSH	238330	Glycine encephalopathy (non-ketotic hyperglycinemia)	605899
36.	GLDC	238300	Glycine encephalopathy (non-ketotic hyperglycinemia)	605899
37.	GLUD1	138130	Hyperinsulinism-hyperammonemia syndrome	606762
38.	GNMT	606628	Glycine N-methyltransferase deficiency	606664
39.	HADH	601609	3-hydroxyacyl-CoA dehydrogenase deficiency (SCHAD deficiency, formerly)	231530
40.	HADHA	600890	Long-chain 3-hydroxyacyl-CoA dehydrogenase deficiency Mitochondrial trifunctional protein deficiency	609016 609015
41.	HADHB	143450	Trifunctional protein deficiency	609015
42.	HLCS	609018	Multiple carboxylase deficiency/holocarboxylase synthetase deficiency	253270
43.	HMGCL	613898	3-hydroxy-3-methylglutaryl-CoA lyase deficiency	246450
44.	HMGCS2	600234	HMG-CoA synthase-2 deficiency	605911
45.	HPD	609695	Tyrosinemia type III	276710
46.	IVD	607036	Isovaleric acidemia	243500
47.	MAT1A	610550	Methionine adenosyltransferase I/III deficiency	250850
48.	MCCC1	609010	3-Methylcrotonyl-CoA carboxylase 1 deficiency	210200
49.	MCCC2	609014	3-Methylcrotonyl-CoA carboxylase 1 deficiency	210200
50.	MCEE	608419	Methylmalonyl-CoA epimerase deficiency	251120
51.	MLYCD	606761	Malonyl-CoA decarboxylase deficiency	248360
52.	MMAA	607481	Methylmalonic aciduria, type cblA, vitamin B12-responsive	251100
53.	MMAB	607568	Methylmalonic aciduria, vitamin B12-responsive, due to defect in the synthesis of adenosylcobalamin, cblB complementation type	251110
54.	MMACHC	609831	Methylmalonic aciduria and homocystinuria, cblC type	277400
55.	MMADHC	611935	Methylmalonic aciduria and homocystinuria, cblD type (alternative gene symbol C2orf25)	277410
56.	MMUT	609058	Methylmalonic aciduria, mut(0) type	251000
57.	NADK2	615787	2,4-dienoyl-CoA reductase deficiency	616034
58.	NAGS	608300	N-acetylglutamate synthase deficiency	237310
59.	OTC	300461	Ornithine transcarbamylase deficiency	311250
60.	OXCT1	601424	Succinyl CoA:3-oxoacid CoA transferase deficiency	245050
61.	PAH	612349	Phenylketonuria due to phenylalanine hydroxylase deficiency	261600
62.	PCBD1	126090	Pterin-4α-carbinolamine dehydratase deficiency	264070
63.	PCCA	232000	Propionic acidemia	606054
64.	PCCB	232050	Propionic acidemia	606054
65.	PPM1K	611065	Maple syrup urine disease, mild variant	615135
66.	PRODH	606810	Hyperprolinemia, type I	239500
67.	PTS	612719	6-pyruvoyl-tetrahydropterin synthase deficiency	261640
68.	QDPR	612676	Dihydropteridine reductase deficiency	261630
69.	SLC22A5	603377	Carnitine uptake deficiency	212140
70.	SLC25A13	603859	Neonatal-onset type II citrullinemia / Neonatal intrahepatic cholestasis caused by citrin deficiency (NICCD)	605814
71.	SLC25A15	603861	Hyperornithinemia-hyperammonemia-homocitrullinemia syndrome	238970
72.	SLC25A20	613698	Carnitine-acylcarnitine translocase deficiency	212138
73.	SUCLA2	603921	Mitochondrial DNA depletion syndrome 5 (encephalomyopathic with or without methylmalonic aciduria)	612073
74.	SUCLG1	611224	Mitochondrial DNA depletion syndrome 9 (encephalomyopathic type with methylmalonic aciduria)	245400
75.	TAT	613018	Tyrosinemia type II	276600

Statistical analysis

Postmortem reference intervals for dried blood spot amino acids and acylcarnitine profile results were established based on profiles from patients without IEM identifiable in the aforementioned IEM gene panel. Outliers were excluded by the Reed method [19], and the non-parametric method was used to derive the central 95% reference intervals [20]. Statistical analysis was performed using MedCalc® Statistical Software version 20.106 (MedCalc Software Ltd, Ostend, Belgium; https://www.medcalc.org; 2022).

## Results

Patient demographics and autopsy findings

Throughout the study period from 2016 to 2021, a total of 43 infants and children who presented with SUD were referred to the authors’ laboratories, including 19 female and 24 male patients (Table 2 and Table 4 in the Appendices). The majority of patients (72%) were aged between 30 days to 12 months, three (7%) were under one month, and nine (21%) were above one year. Among these patients, 18 (42%) had previously undergone newborn screening for inborn errors of metabolism. The cause of death was ascertained in 12 patients. Details of each case are presented in Table 4 in the Appendices.

**Table 2:** Demographics of infants and children presenting with sudden unexpected deaths referred between 2016 and 2021.

	Infants (≤1 year)	Children (>1 year)	All cases
Number of individuals	34	9	43
Male	18 (53%)	6 (67%)	24 (56%)
Female	16 (47%)	3 (33)	19 (44%)
Median age (range)	Weeks	Years	Weeks
Overall	16.1 (0–43.6)	2.6 (1.3–10.9)	17.9 (0–568)
Male	15.9 (0–43.6)	2.4 (1.6–10.9)	18.1 (0–568)
Female	16.6 (6.4–41.7)	3.0 (1.3–4.6)	17.4 (6.4–239)
Cause of death established			
Yes (metabolic)	0 (0%)	1 (11%)	1 (2%)
Yes (Non-metabolic)	5 (15%)	6 (67%)	11 (26%)
No	29 (85%)	2 (22%)	31 (72%)
Newborn screening or other prior metabolic investigation done			
Yes	16 (47%)	3 (33%)	19 (44%)
No	18 (53%)	6 (67%)	24 (56%)
Specimens available at autopsy			
Dried blood spot	13 (100%)	4 (100%)	17 (100%)
Urine	3 (23%)	2 (50%)	5 (29%)
Whole blood	13 (100%)	3 (75%)	16 (94%)

Metabolic investigation results and positive case

Dried blood spot specimens were received for all patients referred to our laboratories. Urine specimens were available in only six cases, heparin plasma in 24 cases, and muscle biopsy in six cases. The specimens, including blood and urine samples, were collected at the time of autopsy, which ranged from one to three days postmortem. The specimens were stored at -20°C with a desiccator until the time of analysis.

Out of all cases referred, acute decompensation of a metabolic disorder was identified as the cause of death in one female patient (patient 11 in Table 4 in the Appendices). The patient was born full-term to non-consanguineous Chinese parents with an unremarkable perinatal history and past medical history. There was no known family history of metabolic diseases. She had not undergone expanded newborn screening for IEM at birth. She presented at 16 months of age with fever and upper respiratory tract symptoms. She was noted to have on-and-off twitching with loss of consciousness at home before being admitted to the emergency department. On arrival at the hospital, she developed cardiac arrest. The blood glucose meter on-spot showed hypoglycemia. Unfortunately, the patient succumbed despite resuscitation and intravenous glucose infusion. Parainfluenzae group 3 direct immunofluorescence and parainfluenza virus 3 RNA was later confirmed positive on nasopharyngeal swabs. Urine metabolic profiling detected significant dicarboxylic aciduria, including glutaric acid, 2-hydroxyglutaric acid, adipic acid, ethylmalonic acid, and methylsuccinic acid, along with marked hyperexcretion of suberylglycine, hexanoylglycine, isovalerylglycine, isobutyrylglycine. Dried blood spot metabolic autopsy was remarkable for a very low level of free carnitine than expected for postmortem specimens. The plasma acylcarnitine profile showed generalized elevations of C4 to C18 acylcarnitines. Next-generation sequencing with the aforementioned IEM gene panel revealed two heterozygous missense variants in the electron transfer flavoprotein dehydrogenase (*ETFDH*) gene, i.e., NM_004453.4:c.1601C>T p.(Pro534Leu) and NM_004453.4:c.1669G>A p.(Glu557Lys), which were also identified by Sanger sequencing with compound heterozygosity of the two variants confirmed by parental genotyping. The first missense variant c.1601C>T is predicted to cause the substitution of proline at residue 534 with leucine at the docking site of electron transport flavoprotein within the electron transfer flavoprotein-ubiquinone oxidoreductase domain [21]. The variant has been reported in multiple patients with multiple acyl-CoA dehydrogenase deficiency [21-24]. Significantly reduced ETFDH protein and deficiencies of oxidative phosphorylation complexes II and III in liver homogenate were demonstrated in compound heterozygous patients harboring the variant [21,22]. The variant has an allele frequency of 0.0024% (6/251308) globally but is absent in controls among East Asians according to the Genome Aggregation Database (gnomAD v2.1.1). It is listed as a disease-causing mutation in Human Gene Mutation Database Professional 2022.1 (CM081237) and a pathogenic variant in ClinVar (RCV000483304.1). The second missense variant c.1669G>A is predicted to cause substitution of glutamic acid with lysine at residue 557 within the electron transfer flavoprotein-ubiquinone oxidoreductase domain, with an allele frequency of 0.0008% (2/250922) globally and 0.0054% (1/18392) among East Asians according to the Genome Aggregation Database (gnomAD v2.1.1). The variant is predicted to be damaging (SIFT/PROVEAN/MetaSVM) and probably damaging (PolyPhen-2) according to in silico analyses. It has been listed as having uncertain significance in ClinVar (VCV000971109.2). Overall, the two variants were considered pathogenic and likely pathogenic, respectively. A diagnosis of multiple acyl-CoA dehydrogenase deficiencies was made based on the biochemical and molecular findings and cascade family screening was arranged.

In the remaining 42 patients, dried blood spot metabolic autopsy and/or urine metabolic profiling did not reveal pathological patterns. Next-generation sequencing with the IEM gene panel in the remaining patients identified three heterozygous likely pathogenic or pathogenic variants in two patients (details in Table 4 in the Appendices). The three variants include (1) NM_001085411.3(NADK2):c.944delG p.(Gly315AspfsTer17), (2) NM_000017.4(ACADS):c.136C>T p.(Arg46Trp) in patient 1, and (3) NM_003850.3(SUCLA2):c.90G>C p.(Gln30His) in patient 20. The three involved genes are implicated in (1) 2,4-dienoyl-CoA reductase deficiency, (2) short-chain acyl-CoA dehydrogenase deficiency, and (3) mitochondrial DNA depletion syndrome 5 (encephalomyopathy with or without methylmalonic aciduria), respectively, and all three phenotypes are autosomal recessively inherited. As there were no corresponding clinical and biochemical features identified in these two patients and no other pathogenic variants were detected by the IEM gene panel, the two cases were considered non-IEM. The range of results and reference intervals of acylcarnitine and amino acid profile in postmortem dried blood spot samples in the remaining patients were presented in Table 3.

**Table 3:** Results and reference interval of dried blood spot amino acid and acylcarnitine profile in postmortem samples and living reference subjects. DBS = dried blood spot; TC = total carnitines Short chain (SC) index = (C2 + C3 + C4)/TC Medium chain (MC) index = (C6 + C8 + C10 + C10:1)/TC Long chain (LC) index = (C14 + C14:1 + C16 + C16:1 + C18 + C18:1)/TC Glutaric aciduria type II (GA-II) index = 1000 × C4 × C5 × C8 × C14)/(C0 × C3)

Analyte (µmol/L)	Postmortem DBS range (median) (n = 42)	Reference interval of postmortem DBS samples	Reference interval of DBS in living reference subjects (age >30 days)
Alanine	840–4,621 (2,124)	868–4,584	161–425
Arginine	1.4–536 (11)	1.4–205	3.2–53
Citrulline	9.1–349 (22)	9.1–114	9–32
Glycine	530–2,657 (1,506)	538–2,639	115–270
Leucine/Isoleucine/Alloisoleucine/ Hydroxyproline	323–3,679 (915)	331–3,616	76–219
Methionine	7.7–816 (130)	9.6–427	8–26
Ornithine	158–1,141 (344)	159–1,122	51–136
Phenylalanine	97–1,538 (279)	100–1,511	24–70
Proline	249–2,399 (567)	251–2,364	68–187
Succinylacetone	0.31–1.5 (0.57)	0.31–1.4	0.32–0.48
Tyrosine	25–1,404 (261)	32–1,371	41–111
Valine	173–1,583 (416)	176–1,558	59–193
C0	94–567 (263)	96–556	21–57
C2	15–95 (50)	16–95	4.7–20
C3	0.99–9.3 (3.6)	1.0–9.2	0.5–2
C3-DC/C4OH	0.64–8.5 (2.3)	0.65–5.6	0.04–0.16
C4	2.2–20 (6.8)	2.3–19	0.09–0.29
C4DC/C5OH	0.22–1.2 (0.62)	0.23–1.2	0.13–0.47
C5	0.31–2.5 (0.77)	0.31–1.7	0.06–0.22
C5:1	0.02–0.16 (0.07)	0.02–0.16	0.004–0.012
C5DC/C6OH	0.45–3.5 (1.4)	0.47–3.5	0.05–0.11
C6	0.28–1.9 (0.78)	0.28–1.9	0.02–0.07
C6DC	0.11–1.5 (0.34)	0.11–1.5	0.06–0.13
C8	0.05–0.35 (0.14)	0.05–0.34	0.02–0.09
C8:1	0.01–0.65 (0.12)	0.01–0.64	0.05–0.33
C10	0.02–0.45 (0.08)	0.02–0.44	0.02–0.1
C10:1	0.01–0.1 (0.03)	0.01–0.10	0.03–0.11
C10:2	0–0.03 (0.01)	<0.03	<0.02
C12	0.01–0.48 (0.10)	0.01–0.48	0.02–0.07
C12:1	0.01–0.10 (0.02)	0.01–0.10	0.02–0.05
C14	0.02–0.78 (0.12)	0.02–0.77	0.03–0.16
C14:1	0.02–0.28 (0.06)	0.02–0.28	0.02–0.07
C14:2	0–0.07 (0.01)	<0.07	0.01–0.03
C14OH	0–0.04 (0.01)	<0.04	<0.01
C16	0.01–4.9 (0.80)	0.02–2.6	0.31–1.5
C16:1	0.02–0.48 (0.08)	0.02–0.47	0.02–0.08
C16:1OH	0.01–0.15 (0.03)	0.01–0.15	0.01–0.05
C16OH	0.01–0.11 (0.03)	0.01–0.07	0.01–0.02
C18	0.1–2.8 (0.54)	0.10–2.8	0.16–0.82
C18:1	0.16–3.9 (0.81)	0.16–3.9	0.47–1.79
C18:1OH	0.01–0.16 (0.04)	0.01–0.16	0.01–0.03
C18:2	0.04–0.89 (0.22)	0.04–0.60	0.2–0.56
C18OH	0–0.1 (0.02)	<0.06	<0.01
TC	127–625 (337)	131–619	-
SC index	0.08–0.30 (0.18)	0.08–0.30	-
MC index	0.0019–0.0059 (0.0033)	0.0019–0.0059	-
LC index	0.0015–0.0408 (0.0070)	0.0015–0.0408	-
GA-II index	0.006–1.96 (0.11)	0.006–1.93	-

## Discussion

This is the first local report on metabolic autopsy combining biochemical and genetic approaches. We have reported the range of acylcarnitine and amino acid levels in postmortem dried blood spots from a group of patients who had been genetically confirmed to be unaffected by any of the known IEMs covered by our gene panel. Our findings were similar to the previous reports, which showed a gross increase in all amino acids and acylcarnitines in dried blood spots obtained in the postmortem period [8,9]. Based on the experience of these authors, diagnosis for metabolic disease in the postmortem period based on dried blood spot metabolic profile is still possible by recognizing a marked increase in a particular disease marker relative to the background increase in other amino acid or acylcarnitine species. We, however, encountered practical difficulties with this approach as the number of fold changes in certain analytes relative to the others to be regarded as significant is poorly defined. Furthermore, some IEM may not have a specific diagnostic marker on dried blood spot metabolic profile. For instance, the postmortem dried blood spot profile of the positive case identified in our study showed a markedly low level of free carnitine, but the characteristic elevation of multiple acylcarnitine species was not evident compared with other postmortem samples, as it overlapped with the typical postmortem generalized increases in multiple acylcarnitine species. Even the use of derived indices combining multiple markers used in living patients, such as the glutaric aciduria-II index [25] did not improve the diagnostic performance. This problem was also encountered in the previous fatal multiple acyl-CoA dehydrogenase deficiency case diagnosed by our group [16].

Organic acid analysis is another important pillar in the postmortem diagnosis of IEM. Both urine and vitreous humor have been reported as suitable for organic acid analysis in autopsy settings [1,11]. In our positive case, metabolite characteristics of multiple acyl-CoA dehydrogenase deficiency were identified in the urine specimen. Although urine organic acids and metabolic profiling are powerful investigations, urine specimens are often not available or only obtained in scant volumes at the time of autopsy as exemplified in our cohort of patients. Vitreous humor was also not available for metabolic investigations in our local protocol as the volume is limited in children and especially infants and is often reserved for other important investigations such as toxicological testing.

Genetic and genomic testing is an important adjunct to the above diagnostic modalities from our experience. DNA can be extracted from specimens that are readily available at autopsy and are stable in the postmortem period. Uncertain biochemical findings can be verified with the sequencing findings, while biochemical data can aid in variant interpretation, especially when variants of uncertain significance are encountered. Simultaneous analysis of multiple IEM genes by next-generation sequencing enhances the cost-effectiveness and practicality of this approach, while the use of a targeted gene panel, as opposed to exome sequencing, reduces the number of variants requiring interpretation and further lowers the cost of analysis.

Throughout the five-year study period, our group had only identified one positive case, fewer than that reported in the previous local study which reported five IEM cases throughout a four-year period between 1999 and 2002 [15]. The apparent reduction in the number of IEM cases diagnosed in this setting may be related to the territory-wide provision of expanded newborn screening of IEM introduced in 2015 [4]. Despite the continuous decline in IEM cases among infants and children presenting with SUD is expected, the metabolic investigation is still an essential part of the autopsy, as patients with late-onset or atypical forms of IEM may not be detected at newborn screening.

Finally, there is still a significant proportion of patients in our cohort with cause of death unidentified after thorough postmortem examinations. Our current analysis focused only on metabolic causes of death. Other mendelian diseases that may present with SUD with negative autopsy findings have not been excluded, including cardiac diseases, such as channelopathies and cardiomyopathies, which have been reported to account for 16% to 34% of SUDs in infants and children [26,27]. Future directions to delineate the cause of death in this group of patients would include panel testing for these cardiac conditions, which may hopefully prevent mortalities in affected families.

## Conclusions

We have reported our five-year experience in the metabolic and genetic autopsy for IEM of children and infants who presented with SUD and have defined the reference intervals for plasma acylcarnitines and amino acids in postmortem dried blood spots based on reference subjects genetically confirmed to be unaffected. We have presented one positive case identified by our group, a case of multiple acyl-CoA dehydrogenase deficiency, which was diagnosed based on the urine metabolic profile and genetic testing. Although postmortem dried blood acylcarnitine and amino acid profile had long been used to screen for fatty acid oxidation disorders and other IEM, our case highlights the diagnostic difficulty of interpreting postmortem dried blood spots, especially in the case of multiple acyl-CoA dehydrogenase deficiency. Our study confirmed the importance of metabolic autopsy and the advantages of incorporating both biochemical and genetic testing in this setting.

## Appendices

**Table 4:** Demographic, autopsy, and metabolic investigation results of the 43 infants/children referred for metabolic investigations. NBSIEM = newborn screening for inborn errors of metabolism; IEM = inborn errors of metabolism; FT = full term; NSD = normal spontaneous delivery; CS = cesarean section; AN = antenatal; PN = postnatal; NICU = neonatal intensive care unit *: Age range: Preterm neonatal = the period at birth when a newborn is born before the full gestation period Term neonatal birth = 27 days Infancy 28 days = 12 months Toddler 13 months = 2 years Early childhood = 2-5 years Middle childhood = 6-11 years

Case	Sex	Age range*	Clinical details	NBSIEM done	Family history	Autopsy findings	Biochemical metabolic study findings	IEM panel testing findings	Cause of death
1	M	Early childhood	FTNSD. Unremarkable AN/PN. Developed a flu-like illness the day before found unresponsive on the bed. The carer later admitted to having strangulated the patient	No	Unremarkable	Signs of mechanical asphyxia	Negative	Heterozygous NM_001085411.3(NADK2):c.944delG p.(Gly315AspfsTer17). Variant not found on gnomAD (v2.1.1). Not reported on HGMD or ClinVar. Heterozygous NM_000017.4(ACADS):c.136C>T p.(Arg46Trp) gnomAD (v2.1.1) allele frequency (all populations): 0.016% (40/251496). Pathogenic in silico predictions by MetaSVM, REVEL, PROVEAN, SIFT. Disease-causing mutation on HGMD Professional 2022.1 (CM890001). Likely Pathogenic on ClinVar (VCV000003825.6)	Pressure on the neck
2	M	Infancy	FTNSD. Unremarkable AN/PN. Found unresponsive on the bed by the mother. During resuscitation blood-stained fluid noted from Ryle’s tube	No	Unremarkable	Unremarkable	Negative	Negative	Unknown
3	M	Early childhood	Elective CS for twin pregnancy. Unremarkable AN/PN. Developed fever and flu-like symptoms a week before. Collapsed at the clinic and died before arrival	Yes	Unremarkable	Empyema. Fatty changes noted in the liver	Negative	Negative	Bronchopneumonia with empyema
4	F	Infancy	FTNSD. AN unremarkable. History of eczema. Found collapsed at home on the bed and plastic cover on the face	No	Unremarkable	Equivocal	Negative	Negative	Unascertained
5	M	Infancy	FTNSD. Unremarkable AN/PN. Found unresponsive on the bed by the parents	No	Unremarkable	Anomalous origin of the right coronary artery compressed between the aortic root and pulmonary trunk	Negative	Negative	Coronary artery malformation
6	F	Infancy	FTNSD. Unremarkable AN/PN. Found unresponsive at home by parents	No	Unremarkable	Equivocal	Negative	Negative	Unascertained
7	M	Infancy	CS. Short NICU stay for desaturation. Otherwise normal AN/PN. Found lying prone on the bed and unresponsive	Yes	Sudden death of elder brother at four months	Unremarkable	Negative	Negative	Unknown
8	M	Infancy	FT. Unremarkable AN/PN. Followed up for cutaneous hemangioma on the forehead and leg	No	Unremarkable	Unremarkable	Negative	Negative	Unascertained
9	F	Infancy	FTNSD. History of neonatal jaundice due to ABO incompatibility. AN/PN otherwise unremarkable. Found collapsed at home	No	Unremarkable	Equivocal	Negative	Negative	Sudden infant death syndrome
10	M	Term neonatal	FT. Induced labor due to low predicted birth weight. AN unremarkable otherwise. Found unresponsive on the bed 2 hours after feed	Yes	Unremarkable	Equivocal	Negative	Negative	Unknown
11	F	Toddlers	FT. AN/PN unremarkable. Presented with fever, upper respiratory symptoms, and convulsion. Cardiac arrest at hospital. Nasopharyngeal swab positive for parainfluenzae group 3	No	Unremarkable	Macrovesicular fatty changes in the liver	DBSM: Low free carnitine; plasma acylcarnitines: generalized elevations of C4 to C18-acylcarnitines; UMP: elevation of glutaric acid, 2-hydroxy-glutaric acids, suberylglycine, hexanoylglycine, isovalerylglycine, isobutyrylglycine, and dicarboxylic acids	Compound heterozygous for NM_004453.4(ETFDH):c.1601C>T p.(Pro534Leu) and NM_004453.4(ETFDH):c.1669G>A p.(Glu557Lys). Please refer to the article for a discussion on pathogenicity	Multiple acyl-CoA dehydrogenase deficiency
12	M	Middle childhood	AN/PN unremarkable. Good past health. Contracted hand-foot-mouth disease a few days before death. Noted sudden collapse after breakfast	No	Unremarkable	Extensive infiltration of lymphocytes in the myocardium involving ventricles and conduction system; postmortem large bowel viral culture was positive for enterovirus and coxsackie virus; tracheal swab culture positive enterovirus/rhinovirus	Negative	Negative	Myocarditis
13	F	Preterm neonatal	Emergency CS at 29 + 6 weeks for placental previa and antepartum hemorrhage. PN complicated by intraventricular hemorrhage, atrial septal defect, and patent foramen ovale. Home leave at corrected gestation of 37 weeks and found unresponsive at home	No	Unremarkable	Unremarkable	Negative	Negative	Unknown
14	F	Infancy	FT CS. AN/PN unremarkable. Slept with the mother on the same bed after a meal. Found unresponsive with blood covering the mouth and nose	Yes	Unremarkable	Negative	Negative	Negative	Unknown
15	M	Infancy	FT CS. AN/PN unremarkable. Poor appetite and later found unresponsive by parents	No	Unremarkable	Marked lymphocytic infiltration in the heart, liver, lungs, and kidneys	Negative	Negative	Lymphoproliferative disease
16	M	Infancy	FTNSD. AN/PN uneventful. Found collapsed at home on the bed lying prone	No	Unremarkable	Negative	Negative	Negative	Unascertained
17	F	Infancy	FTNSD. Noted hepatomegaly at 41 days with elevated ALT at 54 U/L. Later resolved. Found unresponsive at home suspected to have fallen from the bed	Yes	Unremarkable	A small patch of subarachnoid hemorrhage in the left frontal lobe	Negative	Negative	Unascertained. Possible suffocation after head injury
18	F	Infancy	FT CS. AN/PN unremarkable. Found unresponsive on the bed by parents	No	Unremarkable	Negative	Negative	Negative	Unascertained
19	M	Infancy	CS at 34 weeks of gestation (twin pregnancy). PN unremarkable. Had diarrhea on the day before being found unresponsive in a supine position with the face and body covered by a blanket	Yes	Unremarkable	Negative	Negative	Negative	Unascertained. Cannot rule out suffocation
20	F	Infancy	FT NSD. AN/PN unremarkable. Diarrhea for a day with a fever. Found unresponsive at home	Yes	Unremarkable	Postmortem bowel viral culture positive for rotavirus	Negative	Heterozygous NM_003850.3(SUCLA2):c.90G>C p.(Gln30His). Not reported on HGMD or ClinVar gnomAD (v2.1.1) allele frequency (all populations): 0.0018% (2/108418). Predicted loss of nearby 5’ donor splice site by SpliceSiteFinder-like, MaxEntScan, NNSplice, and GeneSplicer	Unascertained
21	M	Toddler	AN/PN unremarkable. Good past health. Received a vaccine the day before and developed a fever. Found unresponsive on the bed	No	Unremarkable	Extensive neutrophils in alveoli	Negative	Negative	Pneumonia
22	M	Infancy	FTNSD. AN/PN unremarkable. Recent flu-like symptoms. Found unresponsive on the bed covered with a blanket over the head	Yes	Unremarkable	Negative	Negative	Negative	Unascertained
23	M	Infancy	FT emergency CS for failed induction. AN/PN uneventful. Found unresponsive at home	No	Unremarkable	Negative	Negative	Negative	Unascertained
24	F	Infancy	FT emergency CS for failed induction. Maternal history of methamphetamine use. AN/PN unremarkable. Unresponsive on the bed lying prone and head covered by a blanket	Yes	Unremarkable	Trace methamphetamine found in blood of uncertain significance. Otherwise unremarkable	Negative	Negative	Unascertained
25	F	Infancy	FT CS. AN/PN unremarkable. Found unresponsive on the bed lying supine	Yes	Unremarkable	Negative	Negative	Negative	Unascertained
26	M	Preterm neonatal	Emergency CS at 28 weeks due to premature rupture of membranes. PN complicated by respiratory distress syndrome requiring mechanical ventilation. Extubated on day three and discharged at one month. Found unresponsive on the bed	No	Unremarkable	Negative	Negative	Negative	Unascertained
27	M	Term neonatal	FTNSD. Found unresponsive five hours after feeding	Yes	Unremarkable	Negative	Negative	Negative	Unascertained
28	M	Early childhood	AN/PN unremarkable. Found unresponsive on the bed while sleeping with the mother	Yes	The younger sister passed away at five months old. Cause of death unascertained	Negative	Negative	Negative	Unascertained
29	M	Infancy	FTNSD. Found unresponsive lying prone on the bed with blood in the nose	Yes	Unremarkable	Negative	Negative	Negative	Unascertained
30	F	Infancy	Past medical history unremarkable. Found unresponsive on the bed	Yes	Unremarkable	Negative	Negative	Negative	Unascertained
31	F	Infancy	FT CS. AN/PN uneventful. Found unresponsive in the bed in a supine position	No	Unremarkable	Negative	Negative	Negative	Unascertained
32	M	Preterm neonatal	Preterm. Emergency CS. No AN check-up. Presented with respiratory distress syndrome and low birth weight and discharged after three weeks of treatment. Found unresponsive at home lying prone on the bed while sharing the bed with parents	Yes	Unremarkable	Methamphetamine found in blood of uncertain significance	Negative	Negative	Unascertained
33	F	Infancy	FT CS. AN/PN unremarkable. Found unresponsive on the bed lying supine while sleeping with parents	Yes	Maternal history of previous miscarriage	Negative	Negative	Negative	Unascertained
34	M	Preterm neonatal	AN unremarkable. Premature rupture of membranes and breech delivery at 26 + 5 weeks before arrival at hospital. No spontaneous breathing at birth and certified dead	No	Unremarkable	Negative	Negative	Negative	Unascertained
35	F	Infancy	FTNSD. AN/PN unremarkable. Fever after immunization. Found unresponsive in the crib after a meal	No	Unremarkable	Negative	Negative	Negative	Unascertained
36	M	Infancy	FTNSD. AN/PN unremarkable. Fever and vomiting two days before death	Yes	Mother and brother had congenital heart defects	Head injuries with subdural and subarachnoid hemorrhages; bilateral retina and optic nerve hemorrhage	Negative	Negative	Head injuries with subdural and subarachnoid hemorrhages
37	F	Infancy	FTNSD. AN/PN unremarkable. A physiological murmur noted at birth which subsided. On the day of death, she developed vomiting, shortness of breath, and collapsed	No	Unremarkable	Hypertrophic cardiomyopathy	Negative	Negative	Hypertrophic cardiomyopathy
38	M	Middle childhood	FT. History of autistic spectrum disorders. Sudden death after repeated vomiting	No	Unremarkable	Megacolon and fatty liver	Negative	Negative	Intestinal obstruction due to Hirschsprung’s disease
39	M	Infancy	FTNSD. AN/PN unremarkable. Noted abnormal breathing and became unconscious on arrival at the hospital	Yes	Unremarkable	Congenital diaphragmatic hernia	Negative	Negative	Bowel ischemia due to congenital diaphragmatic hernia
40	F	Infancy	FTNSD. AN/PN unremarkable. Found unresponsive on the bed in the prone position while sleeping with parents	No	Unremarkable	Negative	Negative	Negative	Unascertained
41	F	Early childhood	FT. Good past health. Sudden death after URTI symptoms	Yes	Not available	Negative. No evidence of pneumonia or sepsis	Negative	Negative	Unascertained
42	F	Early childhood	Good past health. Abdominal pain, vomiting, and diarrhea a few days before. Developed irregular breathing and loss of consciousness	No	Unremarkable	Widespread infiltration of inflammatory cells in both ventricles with multiple foci of myonecrosis	Negative	Negative	Myocarditis
43	M	Infancy	FTNSD. History of NNJ requiring phototherapy. Appeared jaundiced for two weeks and appeared irritable for days. Found unresponsive hours after feeding	No	Unremarkable	Focal myocardial disarray noted. Possible liver fibrosis	Negative	Negative	Unascertained
